# A study of the efficacy and safety of plaque psoriasis treatment by TNF‐α and IL‐17A inhibitor biologics in patients who received the inactivated SARS‐CoV‐2 vaccine

**DOI:** 10.1002/iid3.938

**Published:** 2023-07-27

**Authors:** Shuhong Ye, Hong Sun, Zining Xu, Bingyang Xu, Na Wu, Jiawen Wu

**Affiliations:** ^1^ The Second Affiliated Hospital of Xi'an Jiaotong University Xi'An Shaanxi China; ^2^ Xi'an Jiaotong University Medical School in Department of Nursing Xi'an China

**Keywords:** biologics, inactivated SARS‐CoV‐2 vaccine, psoriasis

## Abstract

**Background:**

Vaccination is an important method for the prevention of severe acute respiratory syndrome coronavirus 2 (SARS‐CoV‐2) transmission. There is currently a lack of real‐world clinical data regarding the safety and efficacy of coronavirus disease 2019 (COVID‐19) vaccines with respect to plaque psoriasis treatment involving tumor necrosis factor‐α (TNF‐α) and interleukin‐17A (IL‐17A) inhibitors.

**Methods:**

We longitudinally analyzed 152 patients with plaque psoriasis, 86 of whom received two doses of inactivated COVID‐19 vaccine (either BBIBP‐CorV or CoronaVac). Comparisons were made between patients undergoing treatment with biologics (TNF‐ α inhibitors or IL‐17A inhibitors) or acitretin. Routine blood tests were used to assess safety; the psoriasis area and severity index (PASI) and dermatology life quality index (DLQI) were used to assess efficacy.

**Results:**

After inactivated COVID‐19 vaccination, biologics retained considerable advantages in terms of improving skin lesions (measured by PASI) and quality of life (measured by DLQI), compared with conventional treatment (*p* < 0.05 and *p* < 0.01, respectively). Routine blood tests and hepatorenal function analyses suggested that inactivated SARS‐CoV‐2 vaccines did not alter the safety of biologics treatment (*p* > 0.05).

**Conclusions:**

Inactivated SARS‐CoV‐2 vaccines do not have significant impacts on the safety and efficacy of biologics (TNF‐α inhibitors or IL‐17A inhibitors) in patients with moderate to severe plaque psoriasis.

## Graphical abstract

1. There is currently a lack of real‐world clinical data regarding the safety and efficacy of coronavirus disease 2019 (COVID‐19) vaccines with respect to plaque psoriasis treatment involving tumor necrosis fact or (TNF)‐α and interleukin (IL‐17A) inhibitors. We longitudinally analyzed 152 patients with plaque psoriasis, 2. Inactivated SARS‐CoV‐2 vaccine has no major impact on the safety and efficacy of biologics (TNF‐α inhibitor or IL‐17A inhibitor) in patients with moderate to severe plaque psoriasis.

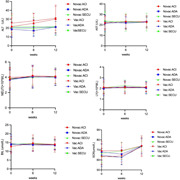

## 1 INTRODUCTION

Since 2019, the destructive effects of coronavirus disease 2019 (COVID‐19) have rapidly spread around the globe. As of November 3, 2022, there were 640 million confirmed cases of COVID‐19 worldwide.^1^ The establishment of a safe and effective vaccine is an important component of efforts to prevent severe acute respiratory syndrome coronavirus 2 (SARS‐CoV‐2) transmission and subsequently achieve control of the pandemic.^2^ Inactivated COVID‐19 vaccines (e.g., BBIBP‐CorV and CoronaVac) have entered phase III efficacy trials and are approved for emergency use in many countries.^3^, ^4^ However, COVID‐19 vaccine hesitancy rates have reportedly reached 50% of the general population in some countries.^5^ Psoriasis is an immune‐mediated inflammatory skin disease that affects approximately 125 million people worldwide.^6^, ^7^, ^8^ The main treatments for moderate to severe plaque psoriasis are biologics, such as anti‐interleukin (IL‐17), anti‐interleukin (IL‐23), and antitumor necrosis factor (TNF‐α), as well as oral agents.^9^, ^10^, ^11^ Compared with nonbiologic systemic therapies, biologics usage has been associated with a lower risk of COVID‐19‐related hospitalization among patients with moderate to severe psoriasis.^12^ Notably, COVID‐19 vaccination has often been hindered by hesitancy based on false beliefs regarding the extension or prevalence of adverse events, potential flare‐ups, and/or inefficient immunization among immune‐mediated skin diseases, such as psoriasis, hidradenitis suppurativa, pemphigus vulgaris, and atopic dermatitis.^13^, ^14^, ^15^, ^16^

Discontinuing, not receiving systematic treatment or refusing COVID‐19 vaccines are very detrimental to psoriasis, not only in physiological and psychological health, long‐term benefits, also are bad for resistance to the COVID‐19. Nonetheless, there are limited clinical data regarding the safety and efficacy of systemic psoriasis treatment after the administration of inactivated COVID‐19 vaccines. Here, we investigated the impact of inactivated COVID‐19 vaccine in patients with psoriasis who were undergoing treatment with biologics agents.

## 2 MATERIALS AND METHODS

### 2.1 Participants

This longitudinal, retrospective, real‐world clinical study included 152 patients (aged 18–70 years) who were clinically diagnosed with moderate to severe plaque psoriasis during the period from October 2020 to June 2022. Patients were excluded if they had hepatitis, tuberculosis, oncological diseases, and/or other systemic diseases; they were also excluded if they had already received COVID‐19 vaccines. On the basis of systemic dermatological therapy, patients were divided into an acitretin group and a biologic (adalimumab or secukinumab) group. Eighty‐six of the 152 patients received two doses of inactivated COVID‐19 vaccine (either BBIBP‐CorV or CoronaVac), these patients were regarded as the vaccinated group, the remaining 66 patients had not received the vaccine were considered as the nonvaccined group.

### 2.2 Clinical protocol

Patients in the acitretin group received acitretin 25 mg once daily, whereas patients in the biologic group received adalimumab 40 mg every other weeks or secukinumab 300 mg every 4 weeks. Before observation, all patients underwent hemocyte tests (neutrophil and lymphocyte counts) and liver and kidney injury markers analyses (serum alanine aminotransferase, aspartate transaminase, total bilirubin, serum creatinine). They were also assessed by an dermatologist to determine their psoriasis area and severity index (PASI) and dermatology life quality index (DLQI) scores. Over the next 3 months, patients who received two doses of an inactivated COVID‐19 vaccine (BBIBP‐CorV or CoronaVac) were regarded as the vaccinated group (as noted above); the remaining patients were regarded as the non‐vaccinated group. The interval between the first and second doses was approximately 4 weeks, all patients had received two doses of the inactivated vaccine by approximately Week 6. Hemocyte tests and liver and kidney injury markers were conducted at 0, 6, and 12 weeks; the PASI and DLQI scores were also reassessed at 12 weeks. Patients were asked to inform the research staff if they experienced any adverse events after vaccination, but their treatments could not be interrupted without consultation.

### 2.3 Evaluation scales and therapeutic agents

The PASI was used to evaluate the severity of skin lesion and the DLQI was used to evaluate impacts on daily life.^17^ Routine blood tests (as noted above) and hepatorenal function analyses (as noted above) were used to assess the safety of inactivated COVID‐19 vaccines.^8^, ^18^, ^19^, ^20^, ^21^ Adalimumab (40 mg) was manufactured by Hisun Biopharmaceutical Co., Ltd. Secukinumab (150 mg) was produced by Novartis Pharma Stein AG. Acitretin (25 mg per pill) was produced by Chongqing Huapont Pharm. CoronaVac (Sinovac Biotech) contained inactivated SARS‐CoV‐2 (3 μg per 0.5‐mL dose), BBIBP‐CorV (Sinopharm/Beijing Institute of Biological Products) contained inactivated SARS‐CoV‐2 (4 μg per 0.5‐mL dose).^22^

### 2.4 Statistical analysis

Statistical analyses were conducted using SPSS software, version 26.0 (IBM Company). Numerical variables are expressed as mean ± standard deviation or median (interquartile range) for parametric and nonparametric variables. Proportions are use to describe categorical data. Indicators describe security (as noted above) used independent sample *t*‐test and the differences in continuous variables used the Mann–Whitney *U* test. Differences in PASI and DLQI response rate were assessed by Kruskal–Wallis one‐way analysis of variance test. *p* < 0.05 is considered as statistically significant.

## 3 RESULTS

### 3.1 Inactivated COVID‐19 vaccines do not alter the therapeutic advantages of biologics

**Figure 1:**
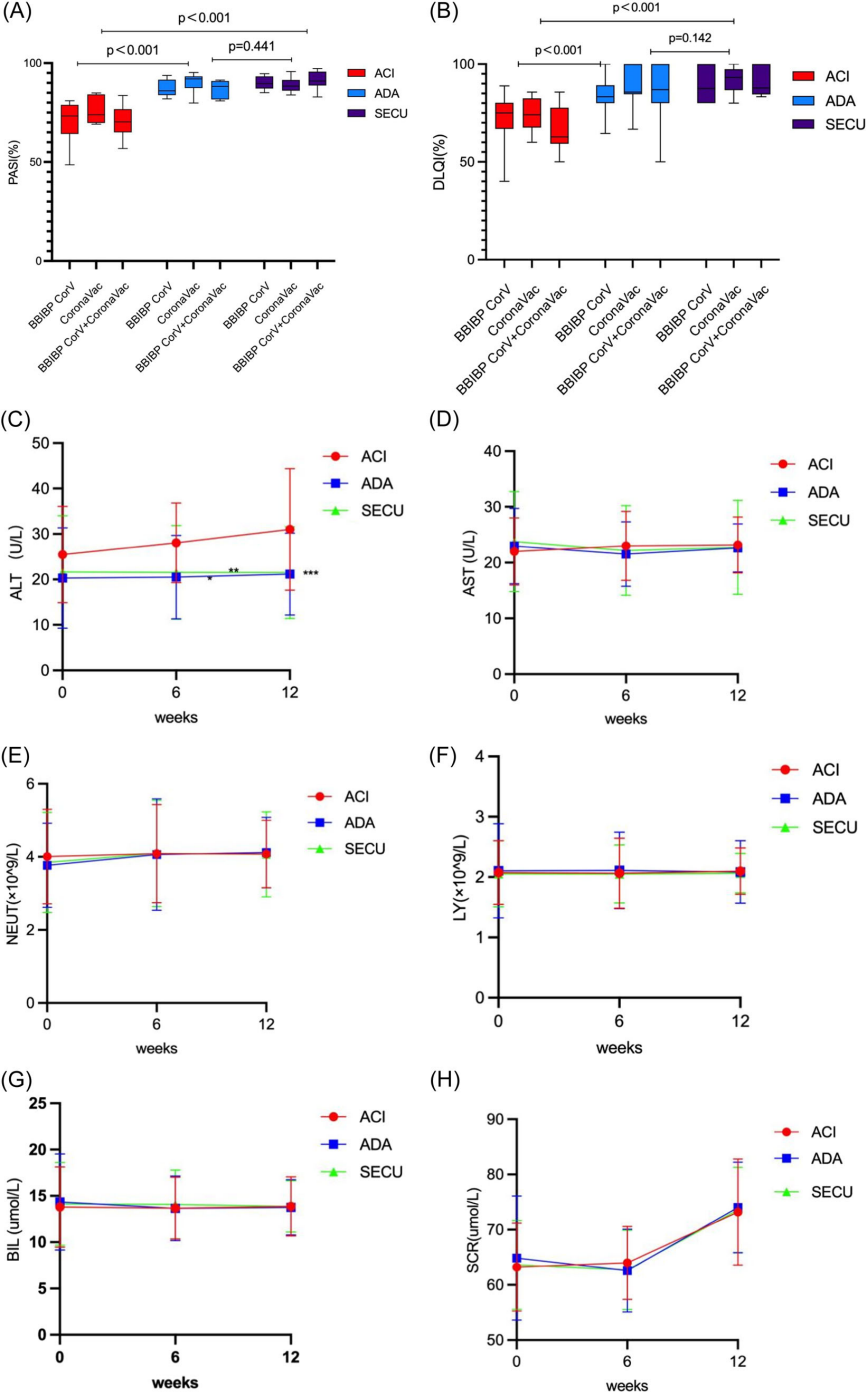
(A, B) Efficacy measures after 12 weeks in vaccine group. (A) Degree of patients PASI response rate. (B) Degree of patients DLQI improvement. *p* < 0.05 represents a statistical difference. There was a difference between the biologics group and the acitretine group in PASI and DLQI response, *p* < 0.001 versus biologics for both (*p* < 0.001 vs. Adalimumab, *p* < 0.001 vs. Secukinumab). Adalimumab and Secukinumab exhibit no significant statistical difference between groups (PASI *p* = 0.441 [A] DLQI *p* = 0.142 [B]). (C–H) Changes in safety indicators over 12 weeks in vaccine group. (C) Alanine aminotransferase (ALT), (D) aspartate transaminase (AST), (E) neutrophil (NEUT), (F) lymphocyte (LY), (G) bilirubin (BIL), (H) serum creatinine (SCR). (E) ALT has a significant increase in acitretine group (ACI), and has a statistical difference between other groups (*p = 0.002 vs. ADA, **p = 0.017 vs. SECU, ***p = 0.900 between ADA and SECU). There was no significant difference in the safety indexes of C–H among the three groups (*p* > 0.05).

PASI and DLQI are commonly used to evaluate the efficacy of psoriasis treatment. Evaluation showed that the biologics group exhibited better performance than the acitretin group in terms of PASI and DLQI response rate (*p* < 0.001 vs. biologics for both) (Figure 1A,B). The difference was not significant between the two biologics subgroups (PASI *p* = 0.11, DLQI *p* = 0.087). Safety throughout the study was evaluated by measuring the concentration of serum ALT, AST, BIL, SCR, and hemocyte NEUT, LY, ALT was elevated in the acitretin group, and showed significant statistical difference with the adalimumab and secukinumab groups (*p* = 0.02 vs. ADA, *p* = 0.17 vs. SECU) (Figure 1C), other security markers did not reveal significant differences among the three groups (*p* > 0.05) (Figure 1D–H).

### 3.2 Effectiveness and safety advantages of biologics

**Figure 2:**
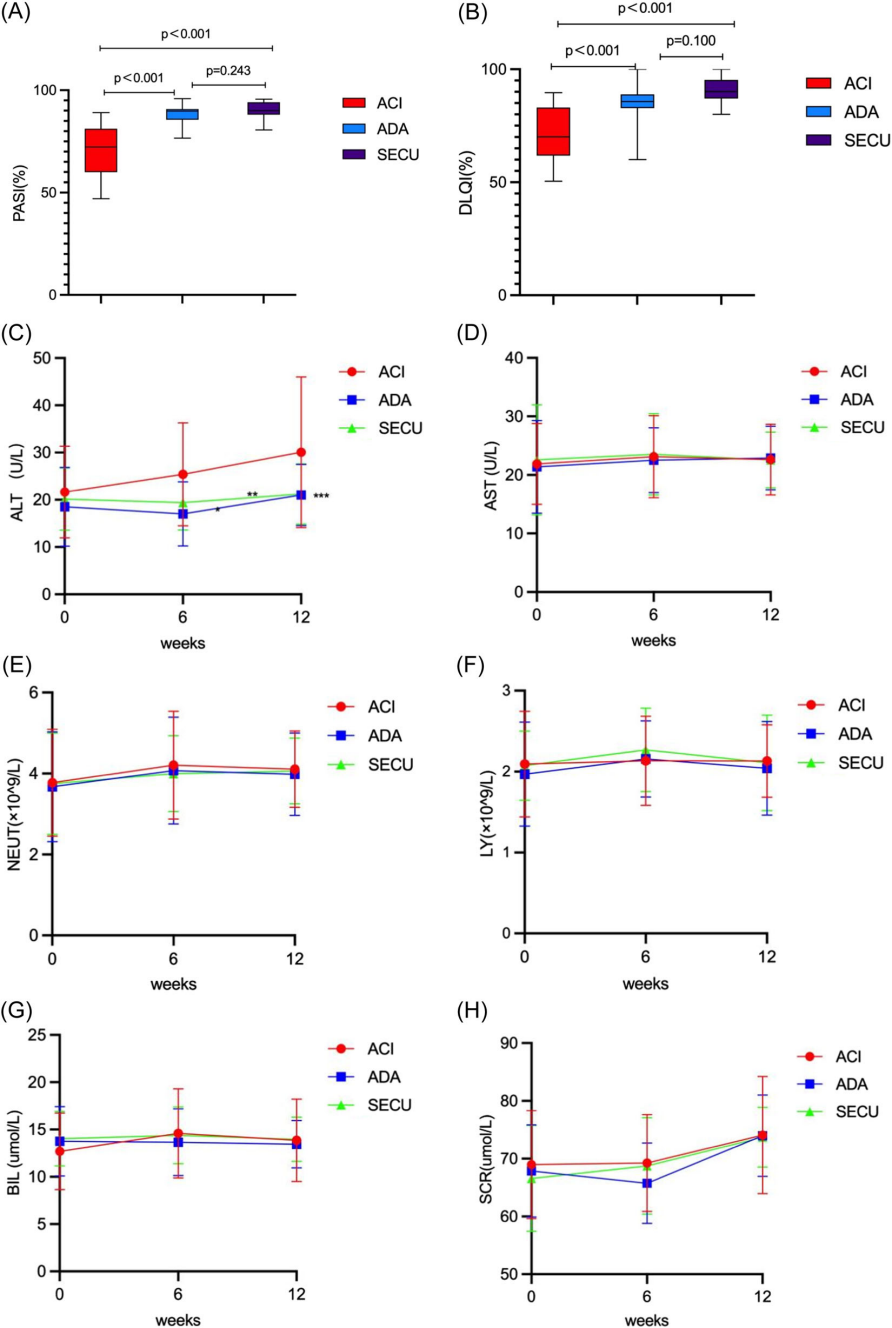
(A, B) Efficacy measures after 12 weeks in nonvaccine group. (A) Degree of patients PASI response rate. (B) Degree of patients DLQI improvement. Response in PASI and DLQI, *p* < 0.001 versus biologics for both (*p* < 0.001 vs. ADA, *p* < 0.001 vs. SECU). Adalimumab and Secukinumab exhibit no significant statistical difference in PASI and DLQI (*p* = 0.243 [A], *p* = 0.100 [B]). (C–H) Changes in safety indicators over 12 weeks in nonvaccine group. (C) Alanine aminotransferase (ALT), (D)aspartate transaminase (AST), (E) neutrophil (NEUT), (F) lymphocyte (LY), (G) total bilirubin (BIL), (H) serum creatinine (SCR). (E) ALT has a significant increase in acitretine group (ACI), *p* < 0.05, has a statistical difference between other groups (*p = 0.01 vs. ADA, **p = 0.031 vs. SECU, ***p = 0.630 between ADA and SECU). There was no significant difference in the safety indexes of C–H among the three groups (*p* > 0.05).

In comparisons with the acitretin group, the adalimumab and secukinumab subgroups exhibited significantly better PASI and DLQI response rate after 12 weeks treatment (*p* < 0.001 vs. ADA, *p* < 0.001 vs. SECU between above two indicators). However, there were no significant differences in PASI or DLQI response rate between the adalimumab and secukinumab subgroups (PASI *p* = 0.243; DLQI *p* = 0.100) (Figure 2A,B). Continuous monitoring of hemocyte and liver and kidney injury markers during the 12 weeks of treatment revealed a significant increase in the ALT level in the acitretin group (Figure 2C) (*p* = 0.01 vs. ADA, *p* = 0.031 vs. SECU), but there were no significant differences between the two biologics subgroups (*p* = 0.630). Other indicators of blood biochemistry and routine blood tests have no significant difference between three groups (*p* > 0.05) (Table 1).

**Table 1:** Demographic of the participants.

Characteristics	Vac group (*n* = 86)			Nonvac group (*n* = 66)			*p*
	Aci	Ada	Secu	Aci	Ada	Secu	
Number	28	31	27	25	20	21	
Age (years)	38.07 ± 12.01	38.35 ± 14.59	34.44 ± 11.8	41.84 ± 15.32	40.20 ± 12.16	36.95 ± 14.72	0.187
Gender							
Male	8	10	8	9	10	8	0.604
Female	20	21	19	16	11	12	

*Note*: *p* value is between Vac group and Nonvac group.

Abbreviations: Aci, Acitretine; Ada, Adalimumab; Nonvac, nonvaccinated; Secu, Secukinumab; Vac, vaccinated.

### 3.3 Inactivated vaccines do not worsen the safety and effect of psoriasis systematic therapy

**Figure 3:**
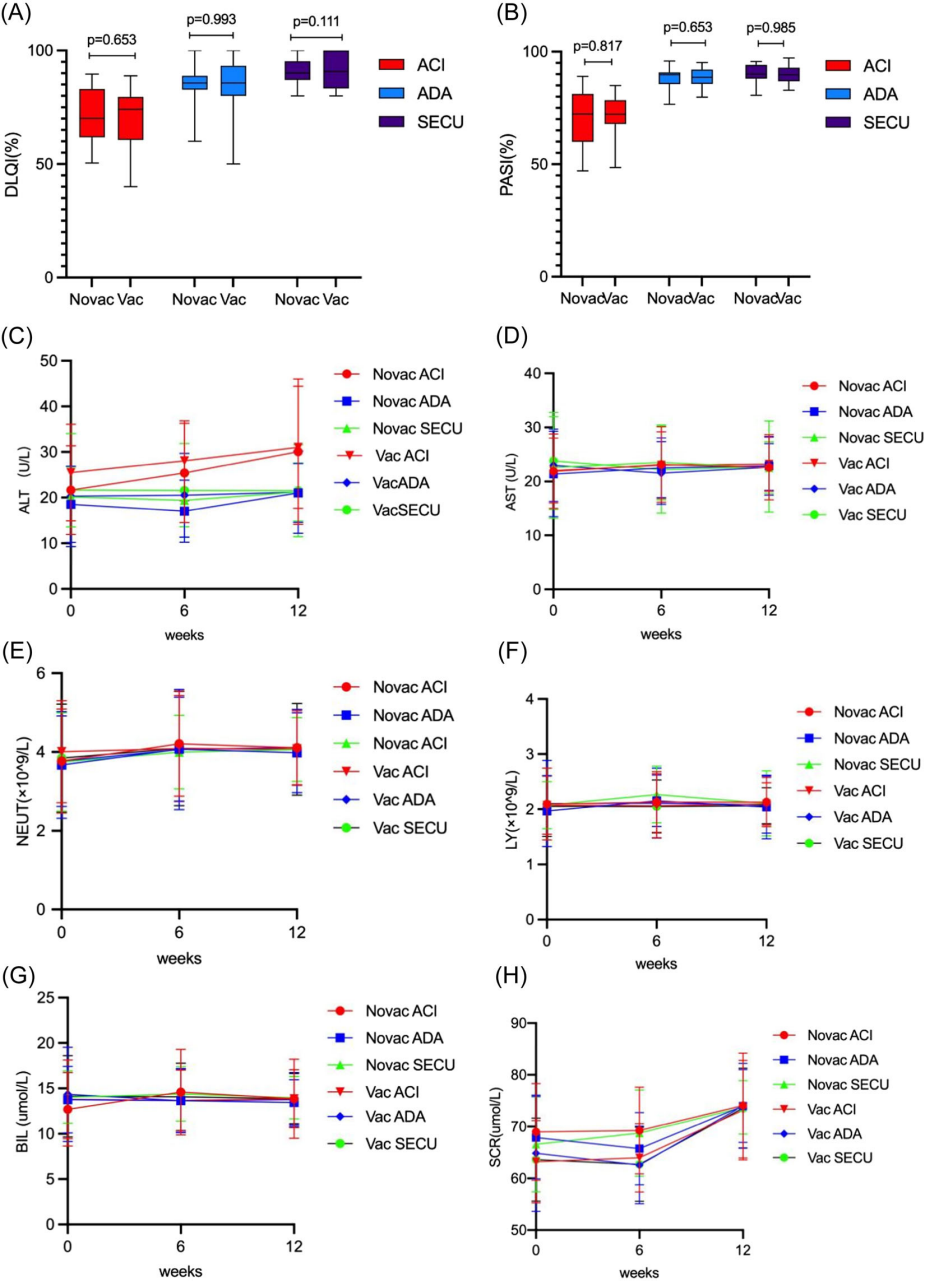
(A, B) Differences in vaccine group and nonvaccine group about the effectiveness assessment of PASI, DLQI. Whether vaccination as a variable, there was no significant difference among the three treatment groups in PASI and DLQI (*p* > 0.05). (C) Alanine aminotransferase (ALT), (D) aspartate transaminase (AST), (E) neutrophil (NEUT), (F) lymphocyte (LY), (G) total bilirubin (BIL), (H) serum creatinine (SCR). (C–H) No significant statistical difference between group vaccine and group nonvaccine (*p* > 0.05).

**Figure 4:**
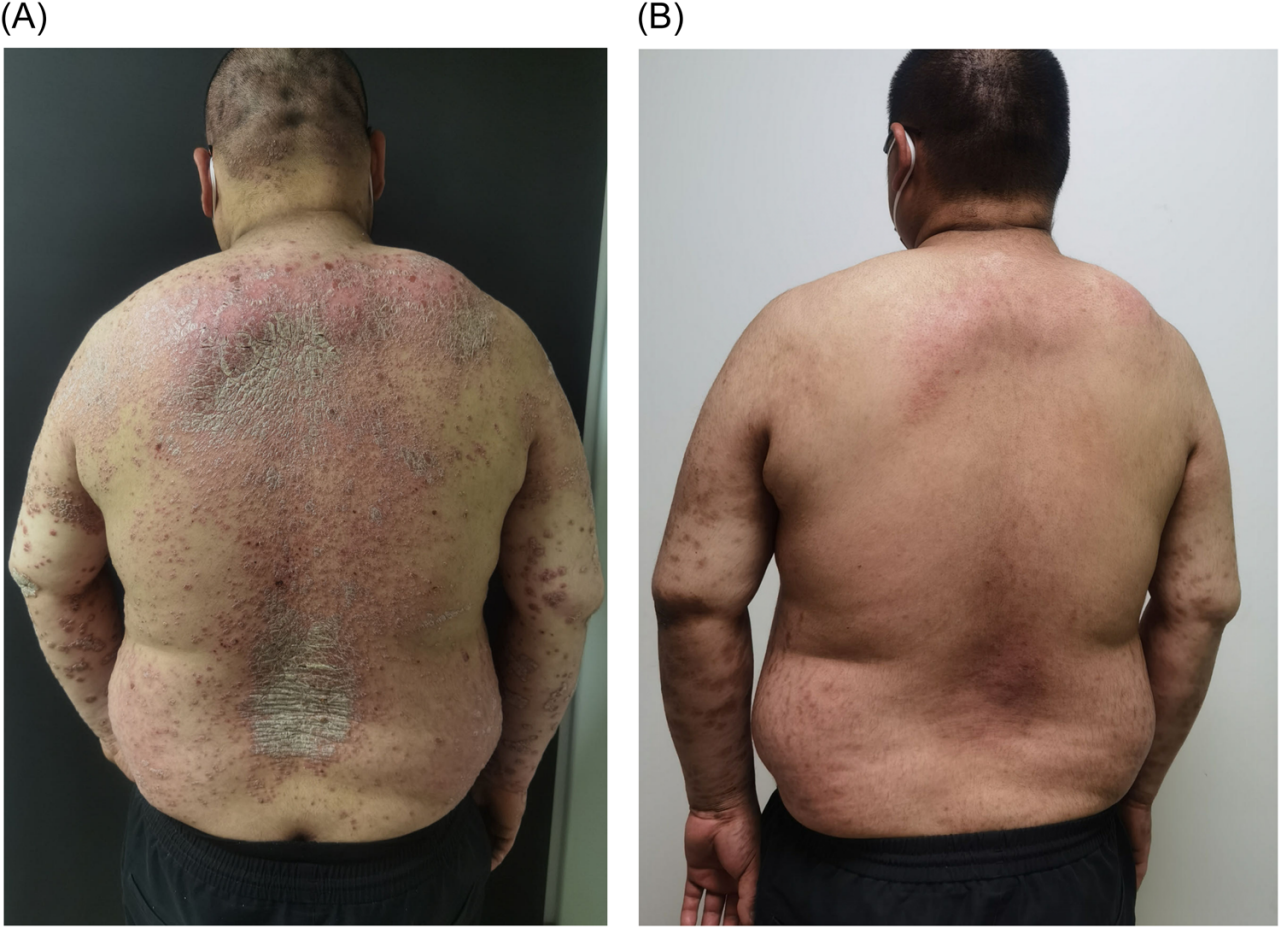
(A) 38‐Year‐old man who regularly received adalimumab 40 mg every week for his psoriasis. He received two doses of COVID‐19 vaccine with the first BBIBPCorV and the second CoronaVac. An expansion of scales and erythema occurred after the second dose of CoronaVac. (A) After being adjusted to secukinumab therapy, his lesions gradually recovered after 12 weeks (B).

We compared the vaccinated group with the nonvaccinated group at the end of Week 12, differences in two groups about the effectiveness assessment of PASI, DLQI rate (Figure 3A,B) and safety detection marker ALT, AST, BIL, SCR, NEUT, LY were not statistically significant (*p* > 0.05) (Figure 3C–H). Additionally, 10 patients experienced transient exacerbation of skin lesions after the first or second dose of inactivated vaccine, but they recovered without any change in treatment regimen. The most serious case involved a 38‐years‐old man who was receiving adalimumab for psoriasis. Before getting vaccine, most of the rash has relieved (PASI = 8). After the first dose of BBIBP‐CorV, there was no significant change in the rash, after received the second dose of CoronaVac, some new erythema began to appear on his upper arm and rapidly spread across his chest and abdomen, he reported a mild cough and sore throat before getting his second vaccine, and expressed a rejection of continuing adalimumab therapy. After being adjusted to secukinumab therapy, his lesions gradually recovered after 12 weeks (Figure 4).

### 3.4 Distinct effects of each inactivated COVID‐19 vaccine on patients with psoriasis

**Figure 5:**
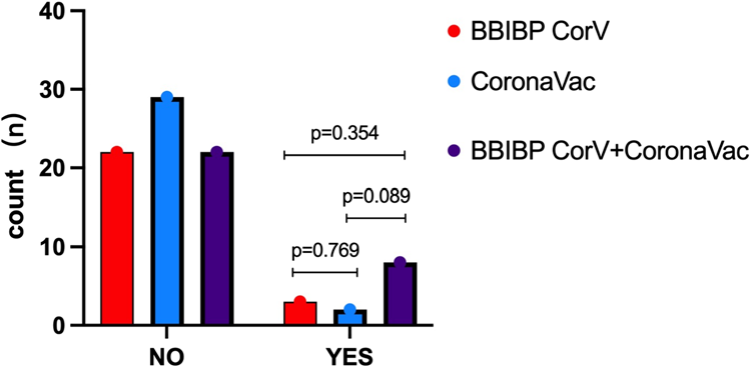
Distinct effects of each inactivated COVID‐19 vaccine on patients with psoriasis. Numbers of people with or without adverse reactions is shown in the graph, NO means without adverse reactions, YES means with adverse reactions, patients are vaccinated in three ways, two dose with BBIBP‐CorV group, two dose with CoronaVac group and have both vaccines administered. three groups showed no significant differences in adverse reactions (*p* > 0.05).

Patients were classified into three groups according to the inactivated COVID‐19 vaccine that they received: two doses of BBIBP‐CorV, two doses of CoronaVac, or one dose of each (i.e., both vaccines). Although more adverse events occurred in the both vaccines group than in the other two groups, the difference was not statistically significant (*p* > 0.05) (Figure 5). These adverse reactions refer to local redness, swelling, and pain rather than serious adverse reactions (Table 2).

**Table 2:** Adverse event analysis of special concern after getting inactivated COVID‐19 vaccine within 12 weeks.

Adverse event *n* (%)	Acitretine (*n =* 53)	Adalimumab (*n* = 51)	Secukinumab (*n* = 48)	*p*
Severe infection	0	0	0	1.000
Opportunistic infection	0	0	0	1.000
Herpes zoster	0	0	0	1.000
Malignant tumor	0	0	0	1.000
Abnormal liver function	3 (5.6)	0	0	0.001
Abnormal renal function	0	0	0	1.000
Dermatoma	0	0	0	1.000
Cardiovascular event	0	0	0	1.000
Hematological Disease	0	0	0	1.000
Anaphylaxis need epinephrine	0	0	0	1.000

## 4 DISCUSSION

In patients with plaque psoriasis who were treated with TNF‐α and IL‐17 A inhibitors, inactivated COVID‐19 vaccines did not alter the original therapeutic advantages of biologics treatment. Moreover, the vaccines did not alter the relative safety of biologics treatments in terms of hepatorenal function or blood test results, compared with acitretin. Finally, no serious adverse events occurred. The most severe case in this study, in addition to different types of vaccines, involved symptoms such as coughing and sore throats that may made the rash worse. Therefore, we conclude that inactivated COVID‐19 vaccines are relatively safe and well‐tolerated among patients with plaque psoriasis. Furthermore, there have no significant impact on adverse events, recovery of skin lesions and improvement of quality of life in psoriasis combined with systemic treatment and homologous inactivated SARA‐CoV‐2 vaccine. In the acitretin group, alanine aminotransferase was elevated regardless of vaccination in some patients; some previous studies have shown that alanine aminotransferase levels are elevated after long‐term administration of acitretin.^23^ Therefore, we speculate that this elevation is a result of acitretin treatment, rather than a result of inactivated COVID‐19 vaccines.

In chronic inflammatory disease such as psoriatic arthritis, hidradenitis suppurativa, alopecia areata, pyoderma gangraenosum, many studies have shown that biologics (e.g., TNF‐α and IL‐17A inhibitors) offer considerable advantages in terms of improving disease status, quality of life, and overall satisfaction.^24^, ^25^, ^26^, ^27^, ^28^ However, in the COVID‐19 pandemic era, flare‐ups, malaise, myalgia, fever, and arthralgia have reportedly occurred after the receipt of the BNT162b2 messenger RNA (mRNA) SARS‐CoV‐2 vaccine among patients receiving topical treatment for plaque psoriasis.^29^, ^30^, ^31^ A few studies have also demonstrated exacerbation of lesions after the receipt of mRNA and adenovirus SARS‐CoV‐2 vaccines among patients with psoriasis who are undergoing treatment with biologics.^27^, ^32^, ^33^ However, no serious adverse events occurred throughout our study, which may be related with the properties of inactivated virus vaccine itself and the comorbidities of the patients. Some clinical studies have confirmed the safety and good tolerability of inactivated vaccines; the overall rates of adverse events were very low, and no deaths were reported.^34^, ^35^, ^36^ In the present study, partial patients experienced transient exacerbation of skin lesions after the first or second dose of inactivated vaccine, whereas three patients reported colds after vaccination, and one patient experienced dietary irritation after vaccination; other patients reported no specific effects. Transient exacerbation of inflammatory responses may be induced by infection, environmental stimuli, or psoriasis progression. However, no serious adverse events occurred, thus, we concluded that inactivated COVID‐19 vaccines have a good safety profile in patients with moderate to severe plaque psoriasis who are undergoing treatment with biologics. Further experiments and data are needed to characterize the mechanisms involving inactivated vaccines in immune‐inflammatory diseases and biologics treatment.

## AUTHOR CONTRIBUTIONS

**Shuhong Ye**: Conceptualization; data curation; formal analysis; investigation; methodology; software; visualization; writing—original draft; writing—review and editing. **Hong Sun**: Conceptualization; formal analysis; methodology; software; visualization; writing—review and editing. **Zining Xu**: Data curation; resources; writing—review and editing. **Bingyang Xu**: Data curation; resources; writing—review and editing. **Na Wu**: Data curation; resources; writing—review and editing. **Jiawen Wu**: Conceptualization; funding acquisition; project administration; resources; supervision; writing—review and editing.

## CONFLICT OF INTEREST STATEMENT

The authors declare no conflict of interest.

## ETHICS STATEMENT

The Ethics Committee of The Second Affiliated Hospital of Xi'an Jiaotong University (Xibei Hospital) approved the study protocol. (The approval number: 2023001) All participants provided informed consent for enrollment in the study.

## Data Availability

Please contact the corresponding author if required.
